# Draft Genome Sequence of Extensively Drug-Resistant *Acinetobacter baumannii* Strain CUAB1 from a Patient in Hong Kong, China

**DOI:** 10.1128/genomeA.00442-15

**Published:** 2015-05-14

**Authors:** Aldrin Kay-Yuen Yim, Jamie Sui-Lam Kwok, Allen Chi-Shing Yu, Alden King-Yung Leung, Hiuus Hiu-Yu Lau, Ting-Fung Chan, Margaret Ip, Stephen Kwok-Wing Tsui

**Affiliations:** a School of Life Sciences, The Chinese University of Hong Kong, Hong Kong, China; b School of Biomedical Sciences, The Chinese University of Hong Kong, Hong Kong, China; c Department of Microbiology, The Chinese University of Hong Kong, Hong Kong, China; d Centre for Microbial Genomics and Proteomics, The Chinese University of Hong Kong, Hong Kong, China

## Abstract

We report the draft genome sequence of an extensively drug-resistant strain of *Acinetobacter baumannii*, CUAB1, isolated from a patient in a local Hong Kong hospital. MIC testing was performed, and genes previously associated with drug resistance were located.

## GENOME ANNOUNCEMENT

The ubiquitous Gram-negative coccobacillus *Acinetobacter baumannii* has attracted attention for the past 30 years due to the emergence of multidrug- or even pandrug-resistant strains. This rapidly emerging pathogen causes infections including bacteremia, pneumonia, meningitis, urinary tract infection, and wound infection (1). To further understand the cause of the drug-resistant phenotype of *A. baumannii*, strain CUAB1 (also known as strain 718532) was isolated from a 70-year-old female patient’s blood in the intensive care unit of a local hospital in Hong Kong, China.

The whole-genome sequence was determined to a depth of 82× using Illumina reads and Velvet 1.1.04 (2) was used for initial *de novo* assembly. Reads were mapped back to the assembled scaffolds to check for misassemblies and erroneous regions were then locally reassembled. Using the published *A. baumannii* ACICU for reference mapping by Burrows-Wheeler Aligner 0.6.1 (3), we identified reads that were unassembled but mapped to the reference sequence. Gaps were then filled by adding the corresponding reference sequence regions into the scaffold. Protein prediction was done using the NCBI prokaryotic genome annotation pipeline (4). The 3.6-Mbp genome was assembled into 16 scaffolds with an *N*_50_ of 706,416 bp and it has 3,426 predicted genes.

MIC tests using broth microdilution were performed for CUAB1. Tests results showed that CUAB1 exhibited high resistance to carbapenems (imipenem and meropenem) and other β-lactams (piperacillin and piperacillin-tazobactam), although it is less resistant to cefoperazone-sulbactam. It is also resistant to aminoglycosides (amikacin, gentamicin, and tobramicin), as well as cephalosporins (cefepime and ceftazidime). Resistance for the fluoroquinolones vary from being resistant to ciprofloxacin to having intermediate resistance to levofloxacin. However, it is sensitive to tigecycline, a glycylcycline antibiotic. CUAB1 was also determined to be resistant to chloramphenicol.

After bioinformatics analysis, we located coding genes related to resistance of the tested drugs that were previously reported. Six β-lactamases (VM83_01790, VM83_06150, VM83_09370, VM83_11515, VM83_12080, and VM83_16400) and two metallo-β-lactamases (VM83_01900 and VM83_14400) are present in the genome. Their mutations or presence may account for resistance to cefepime (5), ceftazidime (6), and piperacillin-tazobactam (7). Genes that were previously reported to be responsible for resistance for aminoglycoside resistance include a resistance-nodulation-cell division (RND) type efflux pump (*adeT* with locus tag VM83_17110), two RND transporters (VM83_17120 and VM83_17125), and two adenine deaminases (VM83_05615 and *adeC* with locus tag VM83_08895) (8). A membrane protein (*carO* with locus tag VM83_12950) might account for carbapenem resistance (9). Moreover, we located DNA gyrase subunit A (VM83_13220) and subunit B (VM83_00010), whose mutations may account for resistance to fluoroquinolone drugs (10, 11). Last but not least, we located three penicillin-binding proteins (PBPs) (VM83_07595, VM83_11570, and VM83_12445) associated with piperacillin resistance (12). We believe the draft genome of CUAB1 will facilitate future genomic analyses of drug resistance mechanisms in *A. baumannii*, given its extensively resistant drug profile and whole-genome annotation.

### Nucleotide sequence accession numbers. 

This whole-genome shotgun project has been deposited in DDBJ/ENA/GenBank under the accession no. JZUF00000000. The version described in this paper is the first version, JZUF01000000.

## References

[1] Fournier PE, Vallenet D, Barbe V, Audic S, Ogata H, Poirel L, Richet H, Robert C, Mangenot S, Abergel C, Nordmann P, Weissenbach J, Raoult D, Claverie JM 2006 Comparative genomics of multidrug resistance in *Acinetobacter baumannii*. PLoS Genet 2:e7. doi:10.1371/journal.pgen.0020007. 16415984 PMC1326220

[2] Zerbino DR, Birney E 2008 Velvet: algorithms for *de novo* short read assembly using de Bruijn graphs. Genome Res 18:821–829. doi:10.1101/gr.074492.107. 18349386 PMC2336801

[3] Li H, Durbin R 2010 Fast and accurate long-read alignment with Burrows-Wheeler transform. Bioinformatics 26:589–595. doi:10.1093/bioinformatics/btp698. 20080505 PMC2828108

[4] Angiuoli SV, Gussman A, Klimke W, Cochrane G, Field D, Garrity G, Kodira CD, Kyrpides N, Madupu R, Markowitz V, Tatusova T, Thomson N, White O 2008 Toward an online repository of standard operating procedures (SOPs) for (meta)genomic annotation. Omics 12:137–141. doi:10.1089/omi.2008.0017. 18416670 PMC3196215

[5] Tian GB, Adams-Haduch JM, Taracila M, Bonomo RA, Wang HN, Doi Y 2011 Extended-spectrum AmpC cephalosporinase in *Acinetobacter baumannii*: ADC-56 confers resistance to cefepime. Antimicrob Agents Chemother 55:4922–4925. doi:10.1128/AAC.00704-11. 21788456 PMC3186995

[6] Opazo A, Sonnevend A, Lopes B, Hamouda A, Ghazawi A, Pal T, Amyes SG 2012 Plasmid-encoded PER-7 beta-lactamase responsible for ceftazidime resistance in *Acinetobacter baumannii* isolated in the United Arab Emirates. J Antimicrob Chemother 67:1619–1622. doi:10.1093/jac/dks087. 22419799

[7] Rice LB, Carias LL, Hujer AM, Bonafede M, Hutton R, Hoyen C, Bonomo RA 2000 High-level expression of chromosomally encoded SHV-1 beta-lactamase and an outer membrane protein change confer resistance to ceftazidime and piperacillin-tazobactam in a clinical isolate of *Klebsiella pneumoniae*. Antimicrob Agents Chemother 44:362–367. doi:10.1128/AAC.44.2.362-367.2000. 10639363 PMC89684

[8] Ardebili A, Lari AR, Talebi M 2014 Correlation of ciprofloxacin resistance with the AdeABC efflux system in *Acinetobacter baumannii* clinical isolates. Ann Lab Med 34:433–438. doi:10.3343/alm.2014.34.6.433. 25368818 PMC4215416

[9] Chiu CH, Lee HY, Tseng LY, Chen CL, Chia JH, Su LH, Liu SY 2010 Mechanisms of resistance to ciprofloxacin, ampicillin/sulbactam and imipenem in *Acinetobacter baumannii* clinical isolates in Taiwan. Int J Antimicrob Agents 35:382–386. doi:10.1016/j.ijantimicag.2009.12.009. 20138741

[10] Miyachi H, Miki I, Aoyama N, Shirasaka D, Matsumoto Y, Toyoda M, Mitani T, Morita Y, Tamura T, Kinoshita S, Okano Y, Kumagai S, Kasuga M 2006 Primary levofloxacin resistance and *gyrA/B* mutations among *Helicobacter pylori* in Japan. Helicobacter 11:243–249. doi:10.1111/j.1523-5378.2006.00415.x. 16882327

[11] Taba H, Kusano N 1998 Sparfloxacin resistance in clinical isolates of *Streptococcus pneumoniae*: involvement of multiple mutations in *gyrA* and *parC* genes. Antimicrob Agents Chemother 42:2193–2196. 9736534 10.1128/aac.42.9.2193 PMC105774

[12] Hakenbeck R, Martin C, Dowson C, Grebe T 1994 Penicillin-binding protein 2b of *Streptococcus pneumoniae* in piperacillin-resistant laboratory mutants. J Bacteriol 176:5574–5577. 8071243 10.1128/jb.176.17.5574-5577.1994 PMC196753

